# Surgical techniques and indications for treatment of adult moyamoya disease

**DOI:** 10.3389/fsurg.2022.966430

**Published:** 2022-08-19

**Authors:** Vincent N. Nguyen, Kara A. Parikh, Mustafa Motiwala, L. Erin Miller, Michael Barats, Camille Milton, Nickalus R. Khan

**Affiliations:** ^1^ Department of Neurological Surgery, University of Tennessee College of Medicine, Memphis, TN, United States; ^2^ Department of Neurosurgery, Semmes-Murphey Neurologic and Spine Institute, Memphis, TN, United States

**Keywords:** bypass, cerebrovascular, indications, moyamoya disease, revascularization, surgery, techniques

## Abstract

Moyamoya disease (MMD) is a chronic, progressive cerebrovascular disease involving the occlusion or stenosis of the terminal portion of the internal carotid artery (ICA) and the proximal anterior and middle cerebral arteries. Adults with MMD have been shown to progressively accumulate neurological and cognitive deficits without treatment, with a mortality rate double that of pediatric patients with MMD. Surgical intervention is the mainstay of treatment to prevent disease progression and improve clinical outcomes. Several different types of bypasses can be utilized for revascularization in MMD, including indirect, direct, and combined forms of extracranial-to-intracranial (EC-IC) bypass. Overall, the choice of appropriate technique requires consideration of the age of the patient, preoperative hemodynamics, neurologic status, and territories most at risk and in need of revascularization. Here, we will review the indications and surgical techniques for the treatment of adult MMD. Step-by-step instructions for performing several bypass variants with technical pearls are discussed.

## Introduction

Moyamoya disease (MMD) is a chronic, progressive cerebrovascular disease involving the occlusion or stenosis of the terminal portion of the internal carotid artery (ICA) and the proximal anterior and middle cerebral arteries. Compensatory enlargement of lenticulostriate and thalamoperforating arteries near the apex of the internal carotid is often seen. The angiographic appearance of these small vessels was first described in 1957 by the Japanese term “Moyamoya,” meaning “puff of smoke” (1–3). The treatment goals of MMD are to augment blood flow and relieve hemodynamic stress on Moyamoya vessels to prevent cerebral ischemia and hemorrhage. Adults with MMD have been shown to progressively accumulate neurological and cognitive deficits without treatment, with a mortality rate double that of pediatric patients with MMD (10% vs. 4.3%, respectively) (4, 5). About half of adult patients present with hemorrhage, whereas nearly all pediatric patients present with ischemia (6).

Several different types of bypasses can be utilized for revascularization in MMD, including indirect, direct, and combined forms of extracranial-to-intracranial (EC-IC) bypass. Indirect bypass does not involve a direct anastomosis. Instead, it relocates vascularized tissue onto the brain's surface to promote angiogenesis utilizing several procedures such as burr holes, pial synangiosis, dural inversion, or omental transposition, among others (7–9). Unlike direct bypass, the indirect bypass only begins to augment cerebral blood flow after angiogenesis has taken place, which may take several months to a year (4, 10). Indirect bypass also eliminates the theorized risk of postoperative cerebral hyperperfusion seen in direct bypass (11).

Direct EC-IC bypass is a procedure first described by Donaghy and Yasargil in 1969 (12) and is commonly utilized in treating adult MMD. Direct bypass includes a direct anastomosis between a donor and recipient artery (typically the superficial temporal artery and middle cerebral artery). Technical variations include 1-donor-1-recipient (“1D1R”), 2-donor-2-recipient (“2D2R” or “Double Barrel”), and a newer technique 1-donor-2-recipient (“1D2R”) (13, 14). Combined bypass offers the immediate improvement in cerebral hemodynamics from direct bypass with the delayed collateralization from indirect bypass (15).

Direct bypass is often preferred in adults because the variants of indirect revascularization are associated with insufficient neo-angiogenesis when compared to children (16). However, controversy remains as to which surgical procedure is superior due to heterogeneous techniques and outcome measures used (17). Prior studies have shown improved functional and symptomatic outcomes (11, 18), angiographic revascularization (17, 19), recurrent stroke (20), and decreased hemorrhage rates (6) when utilizing direct bypass compared to indirect bypass (18) for revascularization in the setting of MMD.

These surgical methods have been compared in a number of studies (17, 21–26). Other meta-analyses have shown better angiographic outcomes with direct or combined techniques vs. indirect bypass (21, 24). Here, we will review the indications and surgical techniques for the treatment of adult MMD. Step-by-step instructions for performing several bypass variants with technical pearls are discussed.

## Indications for surgery

When untreated, both adults and children with MMD suffer from progressive neurologic and cognitive deficits that may result in morbidity or mortality. Prophylactic therapy to reduce the risk of disease progression is crucial to improve outcomes for these patients (4). Medical therapy in the form of aspirin has demonstrated some benefit in ameliorating the effects of strokes, particularly in the pediatric population when cerebral hemodynamics are relatively preserved, but this does not reduce the risk of subsequent strokes (27–30). Surgical revascularization remains the definitive treatment for MMD by augmenting flow to the hypoperfused brain. Surgery is especially crucial for adult patients presenting with hemorrhage as these patients suffer a greater risk of both morbidity and mortality (31–34). Moreover, surgery is indicated based on cerebral blood flow studies demonstrating compromised hemodynamics, angiographic visualization of spontaneous stenosis or occlusion of Circle of Willis vessels, and evaluation of concurrent ischemic or hemorrhagic symptoms (6, 35, 36). Surgical revascularization improves cerebral blood flow, metabolism, and reduces the incidence of future ischemic and hemorrhagic events (15).

Revascularization surgery should be considered for all patients with ischemic or hemorrhagic symptoms and have evidence of impaired cerebrovascular reserve (37). Asymptomatic patients with documented impaired cerebral hemodynamics and progressive imaging findings should be considered for prophylactic revascularization (38).

Diagnostic workup includes CTA and MRI of the brain and formal catheter angiography to define the stage of disease (1), collateral formation, and size of potential donor vessels (37). Although Suzuki grading may not truly reflect the degree of hemodynamic compromise, most bypass candidates are typically stage II-IV disease (38). Advanced imaging studies such as CT perfusion with acetazolamide challenge or MRI with arterial spin labeling may be useful to determine impaired cerebrovascular reserve. In symptomatic patients with radiographic evidence of ischemia and impaired cerebrovascular reserve despite best medical management, surgical intervention should be pursued (37). (FlowChart 1) In hemorrhagic Moyamoya, bilateral revascularization is recommended based on prior prospective, randomized trials (6, 15) showing effective reduction of adverse hemorrhage rates.

Adjunct diagnostic tools to determine bypass candidacy include transcranial doppler vasomotor reactivity analysis (TCD VMR) (39). Patients inhale an O2/CO2 mixture before and after hyperventilation, while TCD is used to measure their MCA velocities. If their mean change in flow velocity from hypocapnia to hypercapnia is greater than 70%, it is normal, whereas changes less than 35% indicate impaired vasoreactivity.

## Bypass techniques

### Indirect bypass

Indirect bypass for revascularization can be performed by several different methods. The classification of the indirect bypass technique is determined by the tissue type used as the donor source for revascularization.

### Encephaloduroarteriosynangiosis (EDAS): arterial donor tissue

This technique is most commonly used for indirect bypass. The superficial temporal artery (STA) is carefully dissected to separate the vessel from the encasing soft tissues, leaving a galeo-adventitial cuff along the artery. The dura is then opened along the course of the artery and the cuff is sewn down to the pia with interrupted suture (29). Other potential donor vessels include the occipital or posterior auricular arteries, which may be mobilized to revascularize different territories as needed. For instance, the posterior circulation can be revascularized using the occipital artery as the donor for the occipital cortex. The occipital artery's convoluted course and submuscular location proximally make graft harvesting less desirable than STA harvest (40). 40–50% of adult patients who undergo indirect bypass do not develop collateral pathways (29). Revascularization is typically obtained in a small area limited to the cortical surface exposed by the craniotomy, which does not restore regional cerebral blood flow in cases with significant anterior cerebral artery (ACA) involvement. Moreover, collaterals typically take 6–12 months postoperatively to develop, during which time the patient is at risk of stroke (29).

In 1984, Endo et al., described the indirect revascularization technique utilizing multiple burr holes to allow external carotid or middle meningeal artery branches to revascularize the brain through these openings (41). When used in conjunction with dural inversion and periosteal synangiosis, this multiple burr hole technique has been shown to be effective (42). However, this technique is unfavorable in patients with cortical atrophy and a significant distance from the brain surface to the burr hole. The burr holes may close prematurely, preventing adequate revascularization. If the revascularization is unsuccessful, future attempts with other bypass techniques may be more challenging (29).

### Encephalomyosynangiosis (EMS): muscular donor tissue

EMS is a technique that was initially described in 1977 by Karasawa et al., in which the temporalis muscle is placed on the surface of the brain (43). As compared to EDAS, EMS carries an elevated risk of mass effect with temporalis swelling causing subsequent cerebral ischemia and seizures. Some have addressed poor cosmetic results by using only the temporalis fascia as a donor with equal angiographic outcomes to standard EMS (44). Kinugasa et al., describes a combined approach of encephaloduroarteriomyosynangiosis (EDAMS), utilizing both EDAS and EMS to maximize the area of vascularized donor with cortical surface (45).

### Dural donor tissue

The technique of dural inversion was described in 1997 by Dauser et al. In this method of revascularization, a dural flap is incised around the middle meningeal artery (MMA) and inverted, placing the highly vascularized outer surface of the dura directly on the cortical surface (46). Others have described a modified version of this technique in which EDAS is performed in conjunction with split-duro-encephalosynagniosis (split DES), in which the outer and inner layers of dura on either side of the MMA were divided, allowing for both layers to attach to the cortical surface (47).

### Other donor tissue

Another indirect bypass technique first described by Karasawa in 1978 is omental transplantation (48). Secondary to the second surgical procedure of the laparotomy for harvest of the omental flap, there are complications related to the addition of the abdominal procedure introduced by using this method (48). Steinberg's group describes a refined procedure using laparoscopic harvest techniques that is better tolerated than open laparotomy with excellent outcomes in pediatric Moya Moya patients (49).

### Direct bypass

Direct bypass most commonly involves the anastomosis of a superficial temporal artery (STA) branch donor directly to a cortical M4 MCA recipient. Direct bypass is widely used for the treatment of MMD and allows for immediate augmentation of blood flow. While this technique may be more technically challenging and dependent on the availability of adequate recipient and donor vessels, it is also associated with more favorable outcomes compared to indirect bypass in adults (50).

In cases where the STA is unfavorable or unavailable, the occipital artery (OA), posterior auricular, or radial artery interpositional graft can be utilized as well (51). The direct bypass has been shown to be beneficial in hemorrhagic Moyamoya Disease by offloading hemodynamic stress from ruptured Moyamoya vessels (52). Direct revascularization has also been shown to prevent further episodes of ischemic stroke over follow-up periods up to 10 years (53). Complications from direct bypass include hyperperfusion syndrome resulting in transient deficits or intracranial hemorrhage (54, 55). Other complications include bypass occlusion, vasospasm, stroke, or aneurysm formation.

Since the first applications of the EC-IC bypass for ischemic vasculopathy in the 1970s, various novel techniques have been developed. These include single donor single recipient (1D1R), two donor two recipients (2D2R), and single donor two recipients (1D2R) (37, 39). Advancement in pre- and intraoperative imaging has allowed for more nuanced techniques, leading to improved revascularization. A case series done on patients who underwent double-barrel (2D2R) bypasses demonstrated an increase in total flow to the affected hemisphere, along with the added benefits of more options and flexibility compared to the single-barrel (1D1R) approach (37).

### 2D2R

The main advantage with 2-donor-2-recipient bypass is revascularization of more than one territory (i.e., frontal and temporal). Double bypass techniques have shown similar flow rates to traditional high flow bypasses such as radial artery or saphenous vein interposition grafts while avoiding the morbidity associated with additional cervical and graft incisions, tunneling, and risk of hyperperfusion hemorrhage (37, 56). Selected patients with multiple areas of need of flow augmentation would benefit from double bypass techniques. Flow-directed targeted revascularization techniques have been demonstrated with the aid of preoperative CT Perfusion and intraoperative ICG videoangiography (37). Intraoperative flow measurements including indocyanine green and ultrasonic flow probe measurement help determine which territories are most in need.

The disadvantages include poor scalp perfusion with possible impaired wound healing and longer operative times. Satellite incisions over the second donor with pulling the second donor into the primary incision over the primary donor have been designed to reduce the risk of scalp ischemia (37).

### 1D2R

Harvest of a single donor vessel can revascularize two separate territories with the 1-donor-2-recipient technique. Advantages include less dissection for revascularization of two territories compared to a 2D2R bypass. The unused STA branch improves scalp perfusion to reduce potential wound complications, proving particularly useful in patients with diabetes or peripheral vascular disease. The preserved branch can also be saved for future bypass if needed (13, 57). These bypasses can provide up to 50% more flow than a single bypass (39). However, they are technically demanding and require both a side-to-side bypass and an end-to-side bypass (13). Both anastomoses and cortical territories are at risk if a technical error occurs.

### Combined bypass

Combined bypass combines a direct bypass with an indirect bypass. This provides immediate revascularization and protection against stroke through the direct bypass with long term protection through the indirect bypass. Large series have demonstrated the prevention of adverse cerebrovascular events for greater than 10 years postoperatively (15).

Combined revascularization technique involves concomitant direct and indirect bypass. Direct STA-MCA anastomosis with indirect EDAS (28, 35) or EDAMS (45, 58) are the most common techniques. Combined bypass harnesses the advantages of both direct and indirect techniques, optimizing immediate revascularization and induction of delayed indirect collaterals (58–60). Indirect bypass is technically uncomplicated and allows for wide cortical revascularization, but the maximum benefit requires time for vascular collaterals to develop with an increased risk of ischemia or hemorrhage during that time (29, 31). Direct bypass provides immediate restoration of cerebral blood flow and has been shown to reduce the risk of stroke and hemorrhage by 2–3 fold when compared to conservative measures (6). The degree of neovascularization may be more substantial following combined bypass as compared to indirect bypass alone (30, 61).

While combined revascularization is a more complex and time-consuming procedure, low complication rates and favorable clinical outcomes have been consistently reported (26, 35, 58, 60, 62). It is also important to consider interactions between direct and indirect bypass and serial changes over time (27). Favorable collateral neovascularization is associated with advanced disease stage in adults but has been suggested to not be associated with the degree of hemodynamic compromise (27). In contrast, other research suggests that the severity of hemodynamic compromise is directly correlated to collateral vessel development (33). While the longitudinal outcomes of combined revascularization merit continued investigation, the literature advocates for combined bypass as a safe and effective intervention for MMD.

## Surgical technique

### Indirect

Indirect bypass, as compared to direct or combined bypass, is not performed with direct anastomosis. The underlying concept for the various types of indirect revascularization is the transposition of a vascularized tissue source onto the surface of the brain. These vascular tissue sources are dissected with care to maintain their blood supply. A craniotomy or burr holes are made to allow the extracranial blood source to lie on the surface of the brain. The process is then dependent on angiogenesis to revascularize the cortex over the course of several months to a year postoperatively (4).

**Figure 1:**
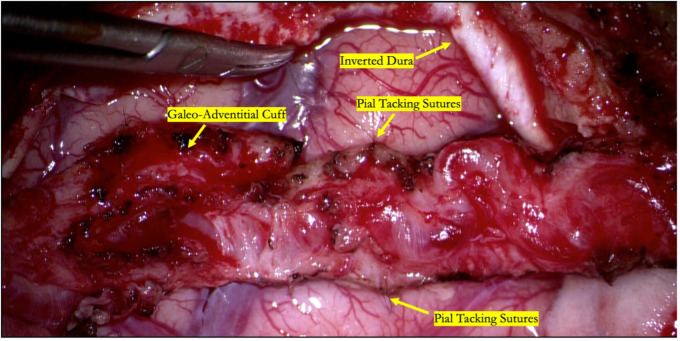
Intraoperative photograph of an indirect STA-MCA bypass (encephaloduroarteriosynangiosis). The parietal branch of the superficial temporal artery is secured to the underlying cortex by suturing the galeo-adventitial cuff to the pia mater using 9-0 nylon suture in an interrupted fashion. Dura is opened to preserve branches of the middle meningeal artery and inverted to augment indirect revascularization.

Most commonly, encephaloduroarteriosynangiosis (EDAS) involves two indirect donors: the superficial temporal artery and the dura mater (Figure 1). A generous galeal-adventitial cuff is harvested with the donor superficial temporal artery to increase the vascularized surface area available to the underlying cortex. The middle meningeal artery is carefully preserved while opening the dura, particularly if there are pre-existing ECA-ICA collaterals on preoperative angiogram. The vascularized dura is then inverted to augment indirect collateralization. The arachnoid is opened widely to reduce any barrier to new vessel ingrowth from the donor sources. The superficial temporal artery's galeo-adventitial cuff is then sewn directly to the pia to maximize contact from the donor tissue to the brain where the arachnoid has been widely opened (63).

### Direct

The most common direct bypass technique involves an STA-MCA anastomosis. Either the frontal or parietal branches of the STA can be used for this purpose, although the parietal branch is more commonly used. STA branches ideally are 1 mm or greater. We have found in some instances that preoperative angiography does not always represent the final vessel size seen intraoperatively.

**Figure 2:**
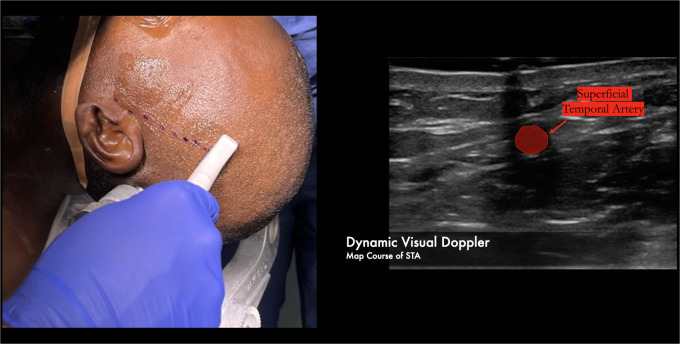
Over 10cm of the parietal branch can be routinely harvested by using a combination of the visual and audio dopplers to map the course of the donor vessel.

We have found success using a visual doppler probe to map the entire length of the superficial temporal artery. This is the smaller “hockey-stick” shaped probe used for radial access in endovascular procedures. The course of the vessel is then confirmed using audio doppler (Figure 2).

**Figure 3:**
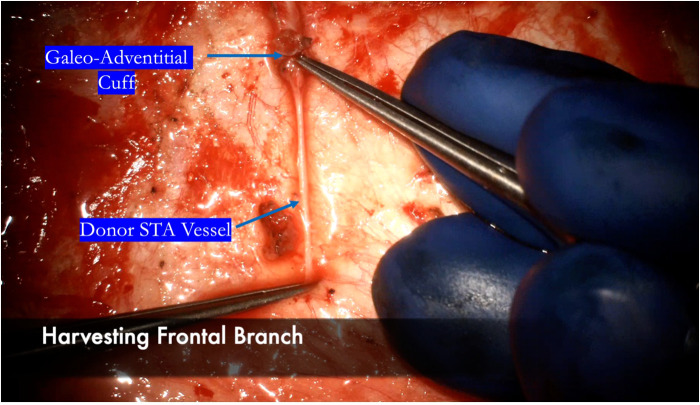
Two fine-tipped bypass forceps are used with the appropriate amount of tension to “strip” the galeo-adventitial cuff off of the donor STA vessel, thereby reducing the resistance around the vessel and maximizing flow through the donor.

**Figure 4:**
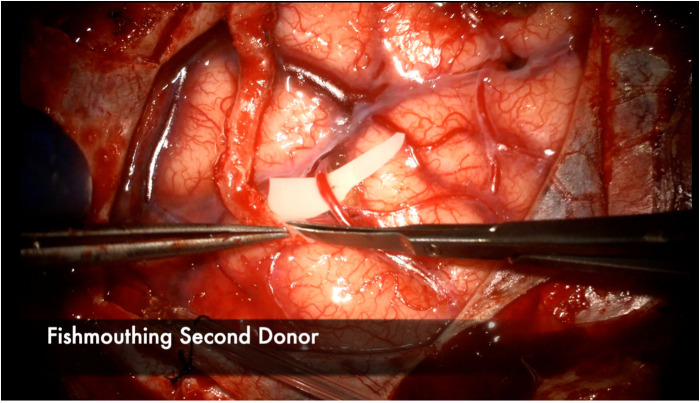
The donor vessel is cut at a 45 degree angle and fish-mouthed to maximize the anastomotic contact area with the recipient vessel. This is performed in this illustration for the second donor vessel in a 2D2R bypass.

**Figure 5:**
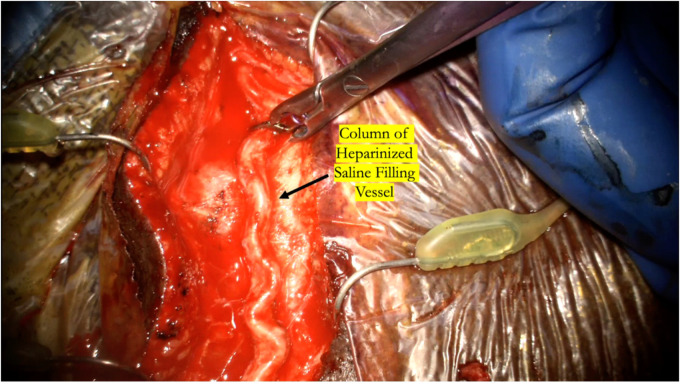
The temporary clip is briefly removed while flushing the distal cut end of the donor vessel with heparinized saline to ensure there is a complete column of heparinized saline that fills the entire vessel all the way proximal to the temporary clip.

We harvest the maximum length possible, typically at least 8–10 cm which gives us multiple bypass options to choose from depending on the available recipient vessels exposed. The vessel is then carefully dissected using a combination of a Mosquito hemostat, 15 blade, micro-scissors, and Colorado tip bovie. An alternative technique popularized by Japanese bypass surgeons involves harvesting the vessel with “bipolar cutting” of the branch vessels to completely skeletonize the artery with great efficiency (64). A critical point is to ensure a thorough dissection and lysing of all fascial bands and excess adventitia that may be constricting or kinking the STA (Figure 3). The recipient vessels are cut at their ends at a 45-degree angle and fishmouthed to maximize the anastomotic area (Figure 4). The donor vessel is flushed with a column of heparinized saline that extends proximal to the temporary clip to ensure no thrombus formation occurs (Figure 5).

**Figure 6:**
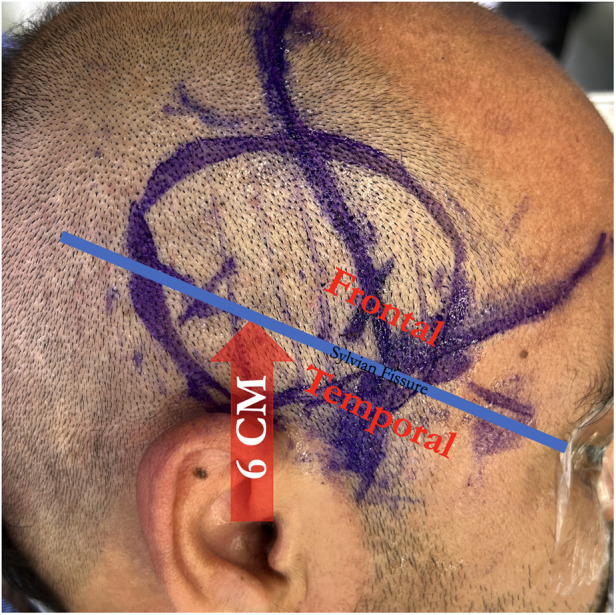
A craniotomy is centered 6 centimeters above the external auditory canal to span the sylvian fissure, giving access to both frontal and temporal M4 recipients.

This is followed by a 3–4 cm frontotemporal craniotomy centered approximately 6 cm above the external auditory canal, which provides exposure of the distal Sylvian fissure and both frontal and temporal lobes (Figure 6). The dura is opened widely in cruciate fashion, preserving the middle meningeal artery branches, and a suitable M4 recipient artery is identified.

**Figure 7:**
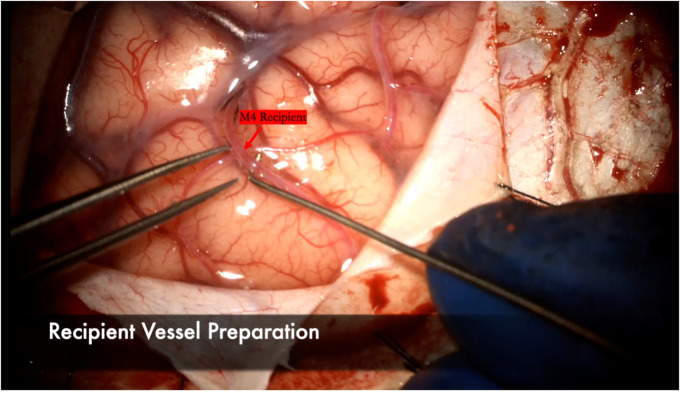
The arachnoid surrounding the M4 recipient is carefully dissected. A microhook is used here to ensure a background can be placed to lift the vessel away from the cortex for bypass. Any small perforating vessels branching from the recipient are sacrificed or secured with mini-clips to ensure no backbleeding and a dry field during the anastomosis.

**Figure 8:**
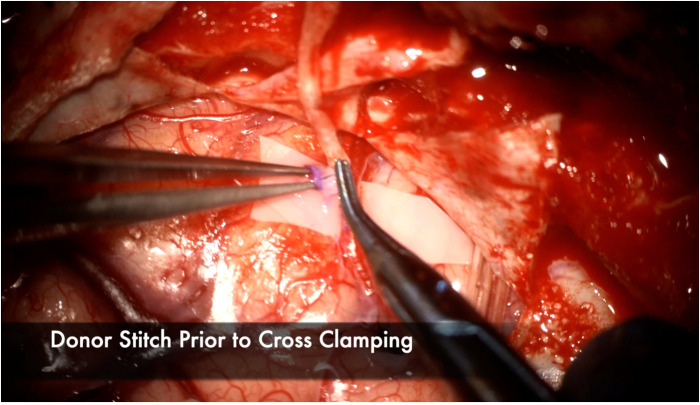
The first stitch is placed in the donor prior to cross clamping to minimize the total ischemia time. It is placed here in an “Out-In” fashion, then in an “In-Out” fashion on the recipient to ensure the stitch is extraluminal. Note the atraumatic technique of placing the bypass forceps gently intraluminally to facilitate throwing the stitch without injuring the intima.

**Figure 9:**
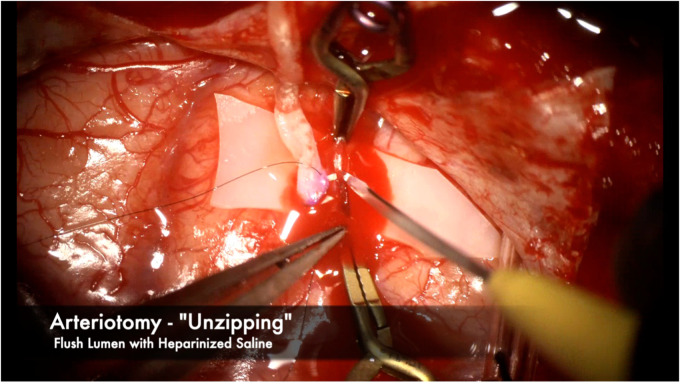
We prefer using an “unzipping” technique with an arachnoid knife to ensure a single, clean arteriotomy line without any jagged edges. The near end of the recipient vessel is gently held with bypass forceps while the knife cuts away from the operator in one clean cut. Heparinized saline is then flushed to thoroughly clean the vessel lumen of any blood and debris.

The recipient M4 artery is thoroughly prepared by isolating small branch vessels with mini-clips or sacrificing them to the minimum extent possible (Figure 7). This step is critical to avoid back bleeding into the recipient vessel during cross-clamping, which can lead to thrombus formation. The anastomosis is prepared by throwing the first stitch into the donor vessel prior to cross-clamping to minimize cross-clamp time (Figure 8). Care must be taken to throw the stitch in an “out-in, in-out” fashion to keep the knot extraluminal. The recipient vessel arteriotomy is best performed with an arachnoid knife to ensure a clean, continuous cut without any jagged edges (Figure 9).

**Figure 10:**
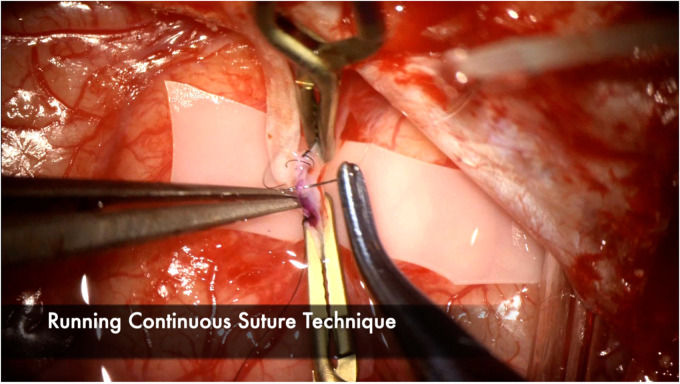
Continuous suture technique is demonstrated here, with emphasis on tying the sutures loosely, particularly at the end of the suture line to facilitate visualization of the lumen and preventing any backwall stitches. 3–5 throws are usually sufficient to ensure a watertight anastomosis. Interrupted suture technique is another effective option but may increase anastomosis time because each knot needs to be tied and cut. Interrupted technique may be favored with very small vessel diameters or when the anastomosis is under tension.

**Figure 11:**
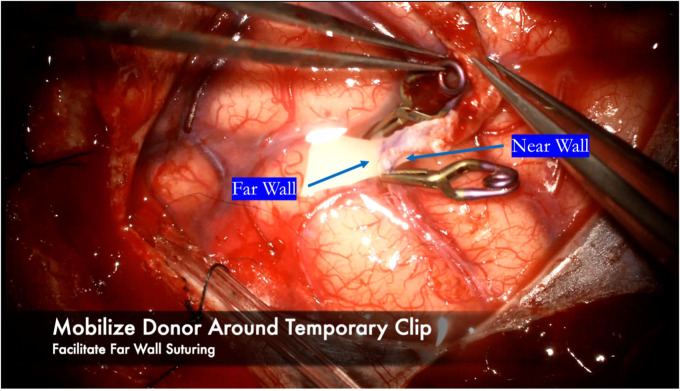
After finishing the near wall suturing, the donor vessel is mobilized around the recipient vessel clips. The clips are used as a buttress to hold the donor vessel in place while sewing the far wall.

**Figure 12:**
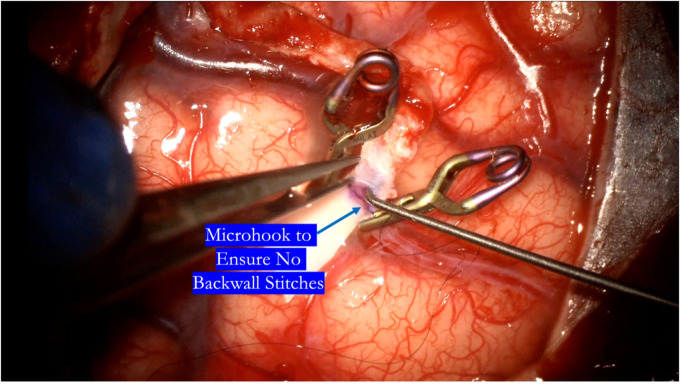
After completing the near wall suturing, a microhook is used to ensure there are no backwall stitches before proceeding with completion of the suture line.

Heel and toe anchor stitches are thrown first to facilitate even suturing on both the far and near sides. Typically 3–5 sutures on each side, not including the heel and toe stitches, are sufficient (Figure 10). Once the near wall has been sewn, the donor vessel is mobilized around the recipient vessel clips to facilitate sewing of the far wall (Figure 11). A microhook is used to ensure there are no backwall stitches before proceeding with suturing of the far wall (Figure 12).

**Figure 13:**
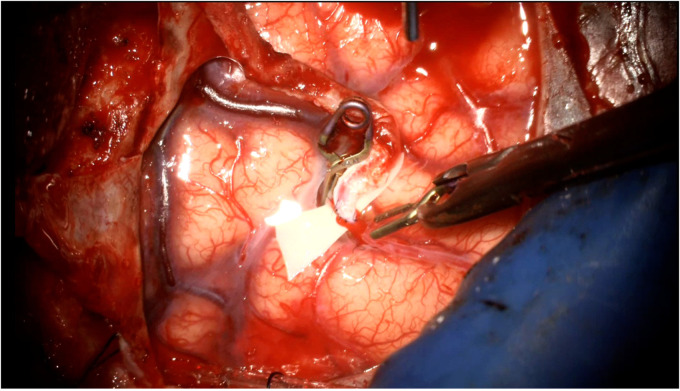
A small amount of backbleeding is welcomed as a healthy sign of a patent anastomosis following removal of the vessel clips. This can be easily controlled with a hemostatic agent such as Surgicel. More vigorous arterial bleeding may require a rescue stitch to control.

**Figure 14:**
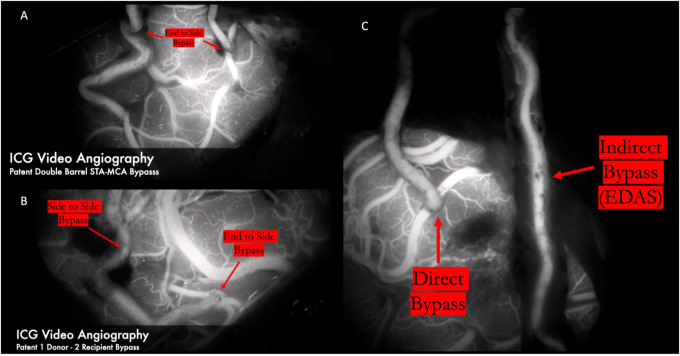
ICG video angiography demonstrates patency of 2D2R bypass (Panel **A**) and 1D2R bypass (Panel **B**), allowing revascularization of both the frontal and temporal MCA territories. Panel **C** demonstrates a combined bypass. First, the frontal branch was dissected clean of all adventitial and fascial constricting bands for a direct anastomosis to the frontal M4 territory. A healthy galeoadventitial cuff was maintained on the parietal branch and tacked down to the pia to indirectly revascularize both the frontal and temporal MCA territories. The direct bypass was seen to fill early and first. The indirect bypass filled in a delayed fashion because of the resistance of the scalp and the galeo-adventitial cuff.

**Figure 15:**
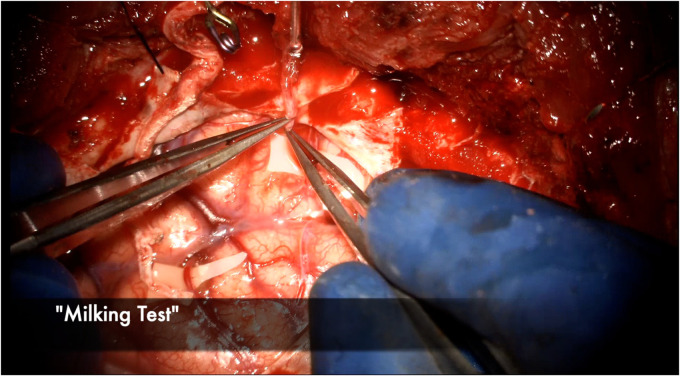
The donor STA is gently “milked” with the micro bypass forceps and then sequentially released, watching for blanching and then immediate refill of the vessel to test for patency.

The anastomosis is backbled by removing the distal temporary clip first. A small amount of backbleeding is evidence of a healthy bypass and easily controlled with a small amount of hemostatic agent (Figure 13). Brisk arterial bleeding that does not respond to these measures may require interrupted rescue stitching. Patency of the bypass can be evaluated using intraoperative indocyanine green (ICG) angiography (Figures 14), micro-doppler, and tactile inspection (50). The “milking test” confirms bypass patency by gently pinching the donor and recipient vessels and watching for an immediate refill (Figure 15).

2D2R bypasses are performed by first harvesting the parietal branch in a distal to proximal fashion. The incision can then be curved anteriorly frontally to harvest the frontal branch in a proximal-distal fashion from within the flap to avoid a forehead scar. Alternatively, a noncontiguous satellite incision can be used to pull the frontal branch in towards the existing incision over the parietal branch (37). Each end-to-side anastomosis performed separately and sequentially. This minimizes temporary clipping time to reduce overall ischemia type for each recipient (Figure 14).

1D2R bypasses are reasonable if the STA bifurcation lies below the level of the planned craniotomy (39). 2D2R bypasses are performed more conveniently if the STA bifurcation is above the craniotomy. Once the parietal branch has been completely dissected, the side-to-side bypass is performed first. Both the recipient and donor vessels matched with linear arteriotomies of equal length about twice the diameter of the recipient vessel. Once the side-to-side bypass is complete and the recipient cross clamping clips are removed, a temporary clip is placed on the donor vessel just distal to the first anastomosis to allow perfusion of that territory. The second bypass is then performed in end-to-side fashion (Figure 14).

### Combined

Combined bypass involves both direct anastomosis and indirect bypass. Most often the procedure involves a superficial temporal artery (STA) to middle cerebral artery (MCA) anastomosis and EDAMS or EDAS indirect bypass. Briefly, both branches of the STA (frontal and parietal) are dissected. Typically the parietal branch is harvested for a longer length and can be disconnected from the scalp as a donor for direct bypass. The remaining STA branch is harvested with a generous galeo-adventitial cuff and pial synangiosis is performed as described previously in the indirect bypass technique section (Figure 14). We recommend performing the direct anastomosis on the territory most at risk based on angiography/perfusion imaging to afford immediate revascularization. The indirect bypass can act as a backup if the direct bypass does not take and for revascularization in a delayed fashion.

## Case presentation

**Figure 16:**
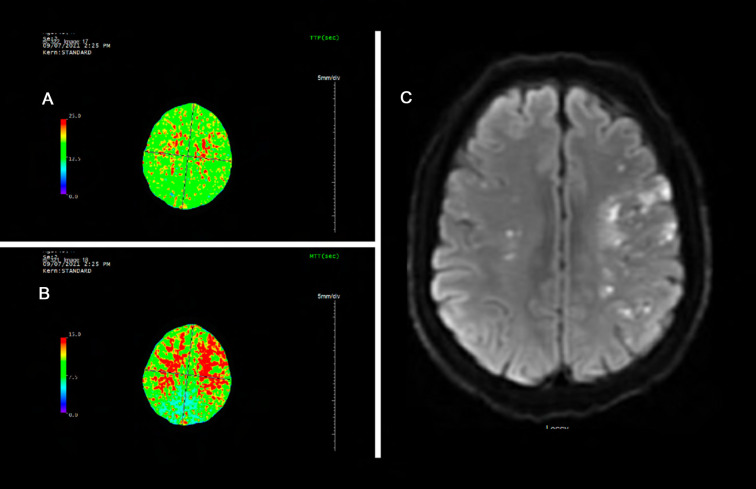
Panel **A** and **B** demonstrate prolonged time to peak (TTP) and mean transit time (MTT) of bilateral cerebral hemispheres. The mean transit time measures 15 seconds through most of the cerebral white matter (normal value ∼6 seconds). This is suggestive of global hypoperfusion. Panel **C** demonstrates bilateral scattered left greater than right infarcts.

**Figure 17:**
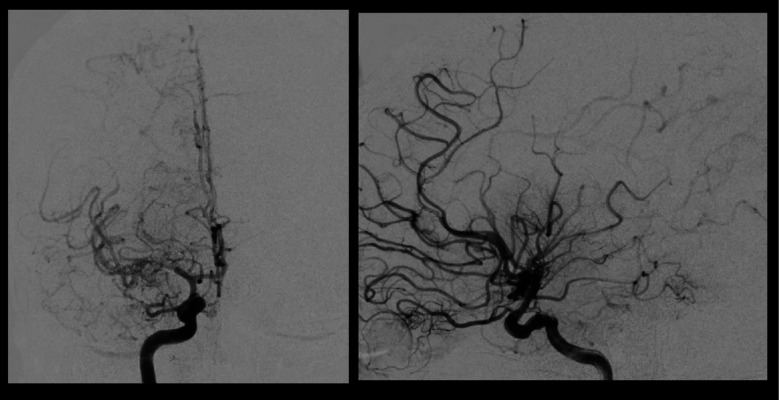
Right sided Suzuki Grade 3 Moyamoya disease is demonstrated on this cerebral angiogram. Smooth tapering of the supraclinoid ICA extending into the proximal MCA with “puff of smoke” lenticulostriate hypertrophy Moyamoya collaterals are visualized. There are some meager ACA-MCA pial collaterals. A large wedge of hypoperfused territory is seen on the lateral projection in the distal MCA territory.

**Figure 18:**
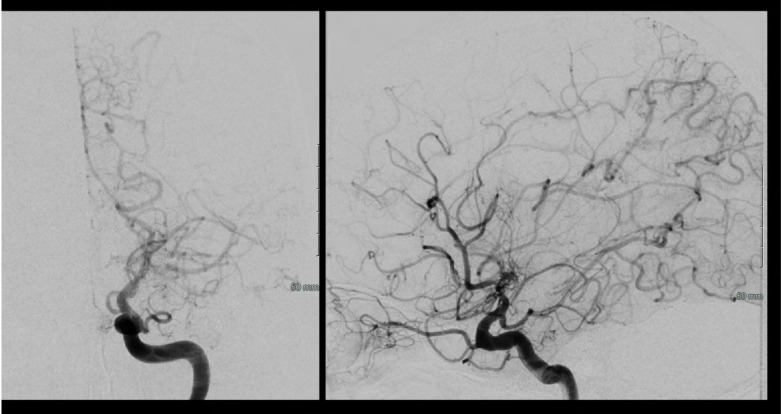
Left sided Suzuki Grade 3 Moyamoya disease is demonstrated on this cerebral angiogram. There is occlusion at the level of the ICA terminus with with hypertrophy of the posterior communicating artery and pial collaterals from the PCA to the ACA territory. His prior angiogram demonstrated filling of bilateral anterior cerebral arteries from this injection, demonstrating significant progression to no further filling of the ACA circulation on this exam.

**Figure 19:**
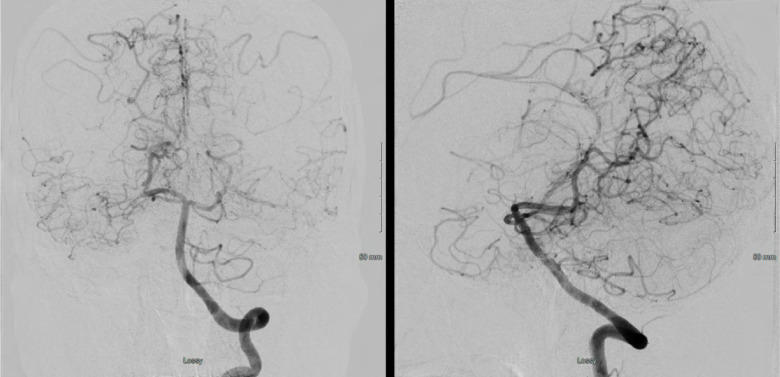
A left vertebral injection demonstrates splenial (PCA) to ACA collaterals and meager lateral temporal and inferior MCA territory pial collaterals.

**Figure 20:**
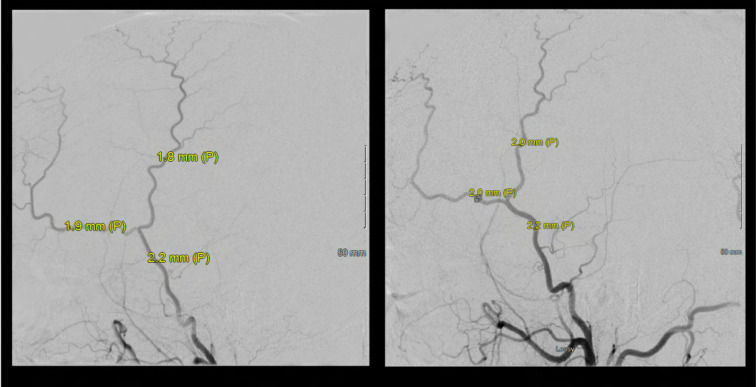
Bilateral ECA injections demonstrate no significant EC-IC collaterals and adequate caliber of the superficial temporal arteries (STA) for direct revascularization. Of note, both frontal and parietal branches of the STA were >0.7mm, which we use as an inferior size limit for direct bypass strategies.

**Figure 21:**
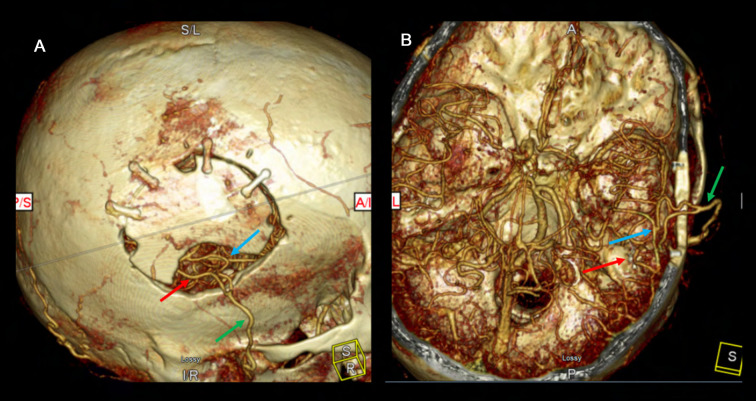
The postoperative CTA demonstrates patency of the right-sided double barrel STA-MCA direct bypass. The 3D reconstruction clearly demonstrates the bony craniectomy made to accommodate the donor graft to keep it free of any compression or kinking. The right MCA territory is well supplied by the STA donors. The green arrow demonstrates the STA main trunk. The red arrow demonstrates the parietal branch of the STA anastomosed to the temporal M4 and the blue arrow shows the frontal branch STA anastomosed to the frontal M4.

**Figure 22:**
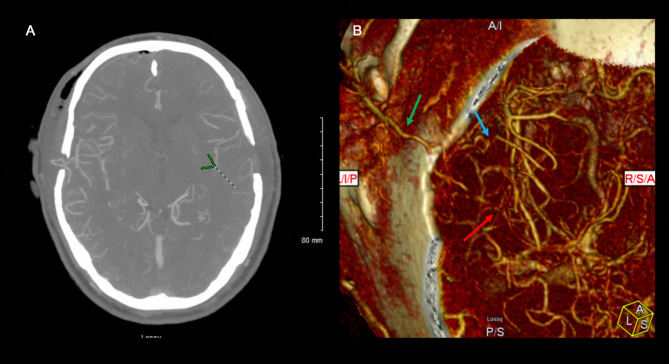
This postoperative CTA demonstrates patency of bilateral STA-MCA bypasses filling both MCA territories robustly. The left sided STA-MCA double barrel bypass is patent (green arrow – main STA trunk; blue arrow – frontal STA to frontal M4; red arrow – parietal STA to temporal M4).

A 46 year old Hispanic gentleman presented with dysarthria and right sided weakness and was found to have bilateral strokes on MRI with the left worse than the right hemisphere. His CT perfusion imaging demonstrated prolonged mean transit time (MTT) and time to peak (TTP) values bilaterally suggestive of global hypoperfusion (Figure 16). He was found to have progression of his Moyamoya disease on angiographic studies, with bilateral Suzuki Grade 3 disease (Figures 17, 18). His posterior circulation offers some meager pial collaterals to the right greater than left MCA territories (Figure 19). His superficial temporal arteries were considered adequate caliber, and there were no significant EC-IC collaterals (Figure 20). Given his active symptomatic presentation and imaging findings consistent with progressive, severe, uncompensated Moyamoya disease, he was offered bilateral cerebral revascularization through “Double Barrel/2D2R” direct bypass (FlowChart 1). Given that he was having ongoing symptoms with recently documented strokes, it was felt that he would most benefit from immediate revascularization through a direct bypass strategy revascularizing the hypoperfused MCA territories. The frontal and parietal branches of the superficial temporal arteries were individually harvested and directly anastomosed to the frontal and temporal M4 territories. Postoperative CTA demonstrates patency of the bypasses (Figure 21, 22). He has maintained on Aspirin 325 mg perioperatively and will continue this for life. He has resolved his preoperative stroke symptoms and has returned to work. This case demonstrates the appropriate imaging workup and decision-making for direct revascularization to prevent further ischemic events in an actively symptomatic adult Moyamoya patient.

## Pearls and pitfalls

Particularly if both frontal and parietal branches are harvested, over-aggressive hemostasis should be avoided as the incision is more likely to necrose given the relative ischemia imparted by the lack of perfusion from the harvested donor scalp vessels. The operative microscope must be utilized for dissection of the donor vessels and for the anastomosis (28, 58). Utilization of the microscope's mouthpiece and/or foot pedals increases surgeon efficiency by keeping both hands in the field while fine adjustments are made to the zoom and focus. The light from a high-powered microscope can easily dehydrate donor vessels, so precautions must be taken to keep the vessels well hydrated throughout the entire surgical procedure. This is of particular importance if a double-barrel bypass is performed, as the second donor vessel can easily dry out during the first anastomosis.

Harvesting of the artery is followed by adventitial stripping and vessel shortening to the minimal necessary length (39). Preoperative assessment for bypass in the setting of MMD should take into consideration vessel flow and resistance in the context of Ohm's law and the law of resistors in series and parallel (39). Measurements of donor cut flow, recipient flow, and final total bypass flow highlight the importance of key surgical steps including adventitia stripping and artery shortening which reduce the total resistance of the system (28, 37). However, care must be taken to avoid too high of flow that may lead to hyperperfusion syndrome, which can occur anywhere from 17 to 50% of cases, especially in patients with a hemorrhagic presentation (39). Hyperperfusion syndrome can cause neurologic deficits that are typically reversible and not explained by other causes, such as hemorrhage or infarction (38). Careful postoperative blood pressure control and judicious perioperative IV hydration maintain hemodynamic balance and help avoid excessive fluctuations that may precipitate hyperperfusion syndrome or infarction (65).

Injection of heparinized saline into the donor vessel throughout the anastomosis with a micro-back autonomous suction placed in the field to wash away blood and debris from the anastomotic site prevents thrombus formation. Wrapping the donor vessel in papaverine-soaked telfa prevents spasm and dehydration (58). It is critical to maintain a dry, bloodless surgical field during bypass to avoid platelet plug and thrombus formation.

These steps optimize bypass patency to avoid occlusion which can be ensured by indocyanine green (ICG) angiography and volumetric flow measurements across the anastomosis site using a microvascular ultrasonic flow probe (39). Meticulous care must be taken during closure to avoid kinking or compression of the vessel by the bone flap, muscle, or skin incision. A tunnel through the bony craniectomy and muscle is often fashioned to ensure the donor vessel is completely free from any compression. Deep galeal stitches are often avoided at the most proximal aspect of the donor vessel to avoid any potential injury or strangulation of the donor. The skin is closed with vertical mattress 3–0 non-absorbable monfilament sutures at the proximal aspect of the incision near the donor, and interrupted suturing for the remainder of the incision.

We administer full dose Aspirin 325 mg at least 3 days before direct bypass surgery in all of our patients and continue it indefinitely postoperatively. The antiplatelet effect is critical to avoid platelet plug/thrombus formation during surgery and maintain bypass patency postoperatively. Small boluses of intravenous heparin may be administered on an as-needed basis if there is a concern for thrombus formation intraoperatively.

## Conclusion

MMD is a unique vascular pathology for which surgical intervention is the mainstay of treatment. While strategies to address this disease vary, well-described methods offer opportunities to prevent disease progression and improve clinical outcomes. Overall, the choice of appropriate technique requires consideration of the age of the patient, preoperative hemodynamics, neurologic status, and territories most at risk and in need of revascularization (29).

## Submission statement

This article is a unique submission, has not been previously published in part or in full, and is not undergoing review with any other journal.
